# Supplementary material for: Gene and allele-specific expression during electric organ ontogeny in African weakly electric fish (Campylomormyrus)
Source: Commun Biol. 2026 Jan 10;9:227. doi: 10.1038/s42003-025-09503-9 (PMC12902082; doi:10.1038/s42003-025-09503-9)
Supplement: Supplementary file 2 — Supplementary Information [file 42003_2025_9503_MOESM2_ESM.pdf]

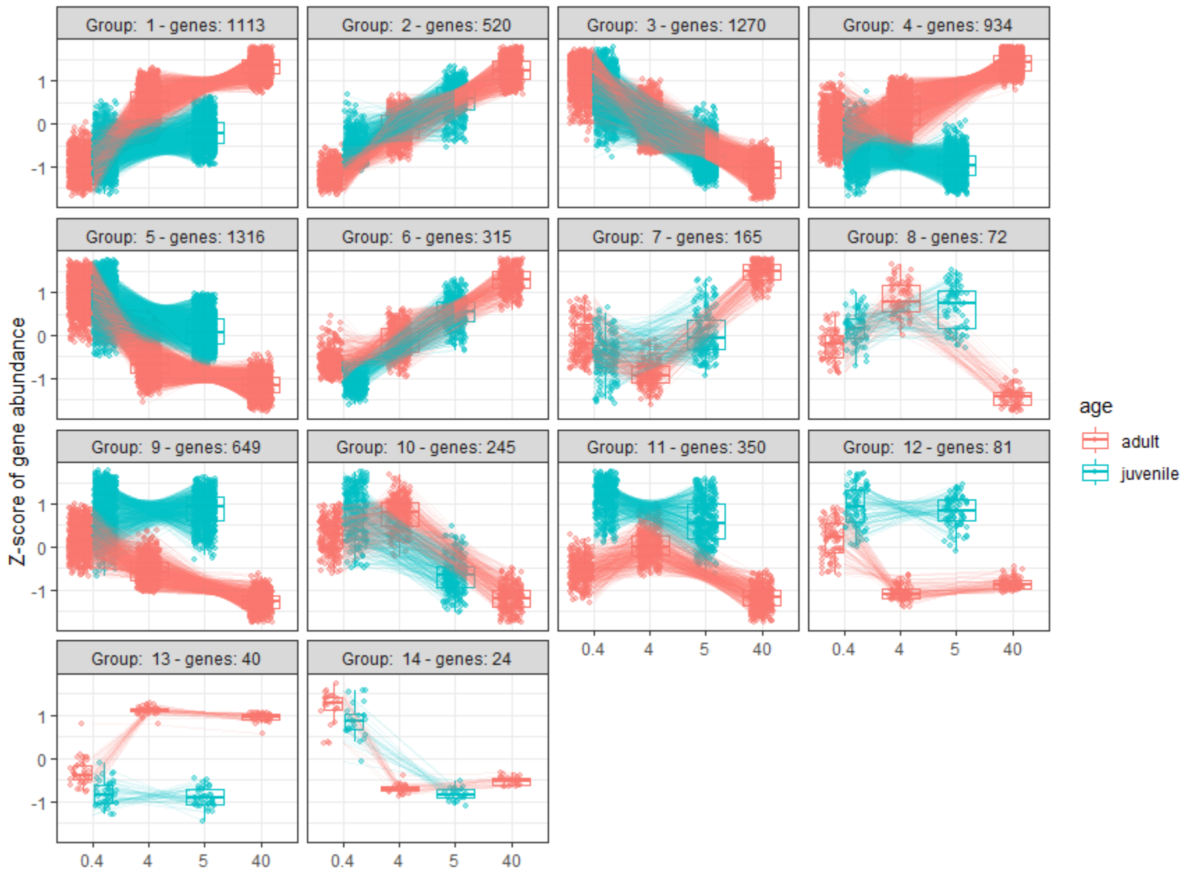

Supplementary Fig. 1. All gene expression patterns that generated by degPatterns.

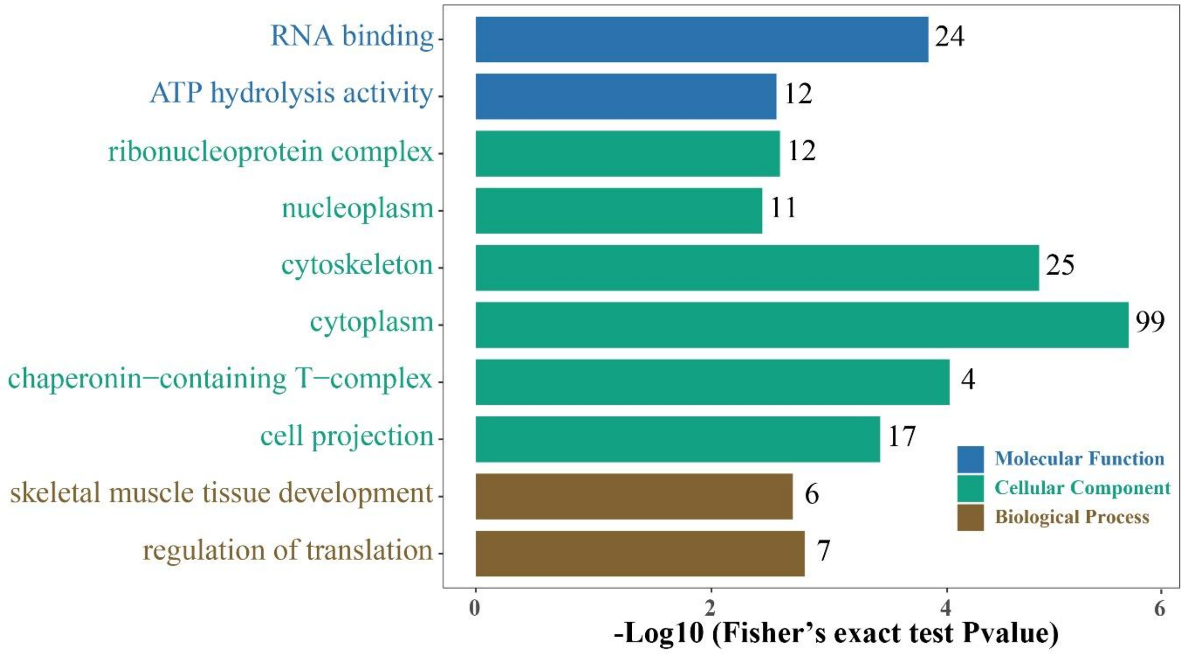

Supplementary Fig. 2. Significantly enriched Gene Ontology terms with Fisher's exact test Pvalue < 0.001 in all genes with fixed bialleles in the hybrids.

Supplementary Table 1. Three significantly enriched Gene Ontology terms with Fisher's exact test Pvalue < 0.05 in group 2, 3, 6 as well as in set A and B.

Term	GO terms	Category	Count	%	Pvalue	Genes	List Total	Pop Hits	Pop Total	Fold Enrichment	Bonferroni	Benjamini	FDR
GO:0005198	structural molecule activity	Molecular Function	5	4.901961	0.001991	EPB41L3A, TUBB5, CLDN11A, CLDN1, MACF1A	89	122	20220	9.311107018	0.284483582	0.334417449	3.34E-01
GO:0003779	actin binding	Molecular Function	6	5.882353	0.020928	EPB41L3A, MICAL2B, MYO5C, SI:DKEY-40C11.2, GSNA, MACF1A	89	362	20220	3.765596871	0.971368636	1	1
GO:0005509	calcium ion binding	Molecular Function	8	7.843137	0.044952	DIPK1B, EGFLAM, PCDH11, MGP, DUOX, SVEP1, MACF1A, PLA2G4AB	89	750	20220	2.423370787	0.999559182	1	1

Supplementary Table 2. All candidate genes in Group 2, 3, 6 as well as in set A and B.

		Data sets							
**Group**	**ID**	**Gene**	**Up regulated in adult EOs (from Cheng et al, 2024)**	**EOD duration candidates in adult EOs (from Cheng et al, 2024)**	**Set A**	**Set B**	**Set C**	**Group 2, 3 or 6**	**Blast**
2	maker-ptg000265l-est_gff_est2genome-gene-6.33-mRNA-1	*KCNJ2*	√	√	√			√	inward rectifier potassium channel 2
3	maker-ptg000950l-augustus-gene-2.9-mRNA-1	*CPNE7*	√	√	√			√	copine-7
3	maker-ptg000119l-snap-gene-11.24-mRNA-1	*CADPSA*	√	√	√			√	calcium-dependent secretion activator 1
2	maker-ptg000610l-snap-gene-7.3-mRNA-1	*SHC4*			√	√		√	SHC adaptor protein 4
3	maker-ptg000922l-snap-gene-6.10-mRNA-1	*DACHD*			√	√		√	dachshund homolog 1
2	snap_masked-ptg000047l-processed-gene-21.0-mRNA-1	*TYRP1*		√	√			√	tyrosinase related protein 1
3	maker-ptg000073l-augustus-gene-32.44-mRNA-1	*PLA2G4AB*		√	√			√	cytosolic phospholipase A2
3	maker-ptg001411l-snap-gene-0.49-mRNA-1	*GALNT7*		√	√			√	polypeptide N-acetylgalactosaminyltransferase 7
3	maker-ptg001903l-augustus-gene-3.70-mRNA-1	*FADS6*		√	√			√	fatty acid desaturase 6
6	maker-ptg000335l-snap-gene-4.58-mRNA-1	*ELP3*		√	√			√	elongator acetyltransferase complex subunit 3
6	maker-ptg001003l-snap-gene-1.56-mRNA-1	*DUOX1*		√	√			√	NADPH thyroid oxidase 1
3	maker-ptg000218l-snap-gene-7.7-mRNA-1	*ADAMTS15*		√	√			√	A disintegrin and metalloproteinase with thrombospondin motifs 15
3	maker-ptg000028l-snap-gene-81.10-mRNA-1	*KCNA7A_1*	√		√			√	potassium voltage-gated channel subfamily A member 7
3	maker-ptg000442l-snap-gene-12.8-mRNA-1	*GRIA3*	√		√			√	glutamate receptor 3
3	snap_masked-ptg000335l-processed-gene-24.16-mRNA-1	*DLGAP2*	√		√			√	disks large-associated protein 2
2	maker-ptg000322l-augustus-gene-12.39-mRNA-1	*RGS4*			√			√	regulator of G-protein signaling 4
3	maker-ptg000150l-augustus-gene-9.7-mRNA-1	*PCDH11*			√			√	protocadherin-11 X-linked
2	maker-ptg000808l-snap-gene-2.21-mRNA-1	*PABPC4*			√			√	poly
2	maker-ptg000734l-augustus-gene-4.95-mRNA-1	*MYO5C*			√			√	myosin VC
2	maker-ptg000135l-augustus-gene-44.3-mRNA-1	*MST1R*			√			√	macrophage stimulating 1 receptor
3	snap_masked-ptg000253l-processed-gene-17.8-mRNA-1	*LSR*			√			√	lipolysis-stimulated lipoprotein receptor
6	maker-ptg001536l-augustus-gene-14.44-mRNA-1	*GPR39*			√			√	G protein-coupled receptor 39
3	maker-ptg000817l-augustus-gene-4.158-mRNA-1	*EGFLAM*			√			√	pikachurin
3	maker-ptg000922l-augustus-gene-6.4-mRNA-1	*DACHD*			√			√	dachshund homolog 1
6	maker-ptg000774l-snap-gene-4.87-mRNA-1	*CUNH18ORF21*			√			√	chromosome unknown C18orf21 homolog
3	maker-ptg002131l-augustus-gene-1.56-mRNA-1	*CLDN7*			√			√	claudin-7
3	maker-ptg000457l-snap-gene-3.42-mRNA-1	*ADGRA1*			√			√	adhesion G protein-coupled receptor A1
3	maker-ptg000585l-augustus-gene-3.81-mRNA-1	*ADGRA1*			√			√	adhesion G protein-coupled receptor A1
2	snap_masked-ptg001427l-processed-gene-12.9-mRNA-1	*ADRA2B*			√			√	alpha-2B adrenergic receptor
3	maker-ptg001880l-snap-gene-1.121-mRNA-1	*CYR61*			√			√	protein CYR61
3	maker-ptg001058l-augustus-gene-1.50-mRNA-1	*RYBP*	√	√		√		√	RING1 and YY1-binding protein B
3	maker-ptg000051l-snap-gene-29.0-mRNA-1	*MIPOL1*	√	√		√		√	mirror-image polydactyly 1
3	maker-ptg000744l-snap-gene-3.34-mRNA-1	*CPT2*	√	√		√		√	carnitine O-palmitoyltransferase 2, mitochondrial
2	maker-ptg001737l-snap-gene-0.24-mRNA-1	*CLDN11*	√	√		√		√	claudin-11
3	snap_masked-ptg000009l-processed-gene-3.14-mRNA-1	*PCDH15*		√		√		√	protocadherin related 15
3	maker-ptg000697l-snap-gene-6.107-mRNA-1	*KCNQ5A*		√		√		√	potassium voltage-gated channel subfamily Q member 5
3	maker-ptg001063l-augustus-gene-1.73-mRNA-1	*DNALI1*		√		√		√	dynein axonemal light intermediate chain 1
3	maker-ptg000346l-augustus-gene-0.0-mRNA-1	*DIPK1B*		√		√		√	protein FAM69B
3	maker-ptg000028l-snap-gene-75.37-mRNA-1	*BRSK2*		√		√		√	serine/threonine-protein kinase BRSK2
3	snap_masked-ptg000335l-processed-gene-29.9-mRNA-1	*SOX11*	√			√		√	transcription factor Sox-11
6	snap_masked-ptg000148l-processed-gene-9.21-mRNA-1	*SLC24A2*	√			√		√	solute carrier family 24 member 2
2	maker-ptg001311l-augustus-gene-8.37-mRNA-1	*HMGB3A*	√			√		√	high mobility group protein B3
3	maker-ptg000871l-augustus-gene-4.6-mRNA-1	*CSMD3*	√			√		√	CUB and Sushi multiple domains 3
3	maker-ptg000632l-snap-gene-6.93-mRNA-1	*B3GLCT*	√			√		√	beta-1,3-glucosyltransferase
3	maker-ptg001157l-snap-gene-8.38-mRNA-1	*MYO5B*	√			√		√	unconventional myosin-Vb
3	maker-ptg000027l-snap-gene-33.3-mRNA-1	*ZFYVE28*				√		√	zinc finger FYVE-type containing 28
3	maker-ptg000051l-snap-gene-70.13-mRNA-1	*WDHD1*				√		√	WD repeat and HMG-box DNA binding protein 1
2	maker-ptg001406l-augustus-gene-3.66-mRNA-1	*VTI1A*				√		√	vesicle transport through interaction with t-SNAREs 1A
3	maker-ptg000600l-snap-gene-6.25-mRNA-1	*VKORC1*				√		√	vitamin K epoxide reductase complex subunit 1
3	maker-ptg000001l-augustus-gene-2.53-mRNA-1	*USP44*				√		√	ubiquitin specific peptidase 44
2	maker-ptg000360l-snap-gene-0.29-mRNA-1	*TUBB5*				√		√	tubulin beta chain
2	maker-ptg000904l-augustus-gene-5.247-mRNA-1	*TMEM233*				√		√	transmembrane protein 233
3	maker-ptg000338l-snap-gene-6.187-mRNA-1	*TIMELESS*				√		√	timeless circadian regulator
3	snap_masked-ptg000040l-processed-gene-15.18-mRNA-1	*SVEP1*				√		√	sushi, von Willebrand factor type A, EGF and pentraxin domain containing 1
3	maker-ptg000688l-snap-gene-4.52-mRNA-1	*SNAI3*				√		√	snail family transcriptional repressor 3
3	maker-ptg000347l-augustus-gene-12.33-mRNA-1	*SH3BP5*				√		√	SH3 domain binding protein 5
3	maker-ptg002649l-augustus-gene-0.4-mRNA-1	*SETD5*				√		√	SET domain containing 5
6	maker-ptg001156l-snap-gene-6.104-mRNA-1	*SDR39U1*				√		√	short chain dehydrogenase/reductase family 39U member 1
3	maker-ptg000057l-snap-gene-47.101-mRNA-1	*RNF11*				√		√	ring finger protein 11
6	maker-ptg000240l-augustus-gene-2.23-mRNA-1	*RND3A*				√		√	rho-related GTP-binding protein RhoE
3	maker-ptg000314l-snap-gene-8.31-mRNA-1	*PTPRD*				√		√	receptor-type tyrosine-protein phosphatase delta
3	maker-ptg001220l-snap-gene-3.79-mRNA-1	*PDE6A*				√		√	phosphodiesterase 6A
3	maker-ptg001623l-augustus-gene-5.37-mRNA-1	*OLFCJ1*				√		√	extracellular calcium-sensing receptor
3	maker-ptg000338l-augustus-gene-6.58-mRNA-1	*NR4A1*				√		√	nuclear receptor subfamily 4 group A member 1
3	snap_masked-ptg000028l-processed-gene-96.4-mRNA-1	*NOG2*				√		√	noggin-2
3	maker-ptg000335l-snap-gene-39.28-mRNA-1	*NKX2.2A*				√		√	homeobox protein Nkx-2.2a
3	maker-ptg000235l-snap-gene-8.11-mRNA-1	*NALCN*				√		√	sodium leak channel, non-selective
3	snap_masked-ptg000869l-processed-gene-8.10-mRNA-1	*MYOCD*				√		√	myocardin
2	maker-ptg000184l-snap-gene-6.2-mRNA-1	*MXRA5*				√		√	matrix-remodeling-associated protein 5
2	maker-ptg002310l-snap-gene-0.35-mRNA-1	*MICAL2B*				√		√	protein-methionine sulfoxide oxidase mical2b
2	maker-ptg000138l-augustus-gene-3.94-mRNA-1	*MGP*				√		√	matrix Gla protein
3	maker-ptg000592l-snap-gene-1.62-mRNA-1	*METTL24*				√		√	methyltransferase like 24
3	maker-ptg000799l-snap-gene-14.13-mRNA-1	*MEF2D*				√		√	myocyte enhancer factor 2D
3	maker-ptg001754l-snap-gene-0.0-mRNA-1	*MACF1*				√		√	microtubule-actin cross-linking factor 1
2	maker-ptg001399l-snap-gene-0.80-mRNA-1	*LPL*				√		√	lipoprotein lipase
6	maker-ptg000335l-augustus-gene-16.38-mRNA-1	*LAPTM4A*				√		√	lysosomal-associated transmembrane protein 4A
3	maker-ptg001458l-est_gff_est2genome-gene-14.5-mRNA-1	*KIAA1257*				√		√	KIAA1257 ortholog
6	snap_masked-ptg001226l-processed-gene-1.9-mRNA-1	*HSP70*				√		√	heat shock 70 kDa protein
3	maker-ptg001938l-augustus-gene-0.25-mRNA-1	*HPGD*				√		√	15-hydroxyprostaglandin dehydrogenase [NAD
2	maker-ptg002297l-snap-gene-0.45-mRNA-1	*GSN*				√		√	gelsolin
3	maker-ptg001601l-snap-gene-2.66-mRNA-1	*GRAMD2A*				√		√	GRAM domain containing 2A
3	maker-ptg002632l-snap-gene-3.102-mRNA-1	*FRMPD1*				√		√	FERM and PDZ domain-containing protein 1
3	maker-ptg000187l-augustus-gene-12.53-mRNA-1	*FKBP6*				√		√	FK506 binding protein 6
3	maker-ptg002265l-snap-gene-1.34-mRNA-1	*EML6*				√		√	Syngnathus acus genome assembly, chromosome: 1
3	maker-ptg000051l-augustus-gene-36.18-mRNA-1	*EGLN3*				√		√	egl-9 family hypoxia inducible factor 3
6	maker-ptg001427l-augustus-gene-28.3-mRNA-1	*DOK2*				√		√	docking protein 2
2	snap_masked-ptg000367l-processed-gene-5.230-mRNA-1	*DOCK9*				√		√	dedicator of cytokinesis 9
2	maker-ptg000424l-snap-gene-7.8-mRNA-1	*DDX27*				√		√	DEAD-box helicase 27
3	maker-ptg000136l-snap-gene-4.37-mRNA-1	*COL4A6*				√		√	collagen alpha-6
3	maker-ptg000335l-snap-gene-47.22-mRNA-1	*CNKSR3*				√		√	CNKSR family member 3
6	maker-ptg000160l-snap-gene-11.44-mRNA-1	*CHRND*				√		√	acetylcholine receptor subunit delta mRNA, partial cds
3	maker-ptg001315l-snap-gene-0.23-mRNA-1	*CADM1*				√		√	cell adhesion molecule 1
3	maker-ptg000994l-snap-gene-2.19-mRNA-1	*CACNA2D1*				√		√	voltage-dependent calcium channel subunit alpha-2/delta-1
3	snap_masked-ptg000868l-processed-gene-3.177-mRNA-1	*CACHD1*				√		√	cache domain containing 1
3	maker-ptg001603l-snap-gene-0.37-mRNA-1	*C2CD2L*				√		√	phospholipid transfer protein C2CD2L
3	snap_masked-ptg000068l-processed-gene-18.12-mRNA-1	*BHLHE40*				√		√	class E basic helix-loop-helix protein 40
6	maker-ptg000442l-augustus-gene-28.32-mRNA-1	*ATP13A2*				√		√	ATPase 13A2
3	maker-ptg000307l-snap-gene-1.114-mRNA-1	*ASPG*				√		√	asparaginase
2	maker-ptg000786l-snap-gene-1.61-mRNA-1	*ARPP21*				√		√	cAMP regulated phosphoprotein 21
3	maker-ptg001188l-snap-gene-7.46-mRNA-1	*ARHGAP27*				√		√	rho GTPase-activating protein 27
2	maker-ptg000316l-augustus-gene-4.73-mRNA-1	*ADSS*				√		√	adenylosuccinate synthase
3	maker-ptg000085l-snap-gene-8.23-mRNA-1	*ABHD6*				√		√	abhydrolase domain containing 6
6	snap_masked-ptg000686l-processed-gene-5.15-mRNA-1	*CHST8*				√		√	carbohydrate sulfotransferase 8
3	maker-ptg001619l-augustus-gene-1.271-mRNA-1	*LCP1*				√		√	digestive cysteine proteinase 1
2	maker-ptg000393l-snap-gene-11.3-mRNA-1	*DBN1*				√		√	drebrin
3	maker-ptg001531l-augustus-gene-0.35-mRNA-1	*TRIM39*				√		√	E3 ubiquitin-protein ligase TRIM39
3	snap_masked-ptg000694l-processed-gene-0.1-mRNA-1	*POU4F3*				√		√	POU domain, class 3, transcription factor 4
3	maker-ptg000509l-est_gff_est2genome-gene-1.0-mRNA-1	*PGBD3*				√		√	piggyBac transposable element-derived protein 3
3	maker-ptg000361l-snap-gene-38.44-mRNA-1	*EPB41*				√		√	protein 4.1
6	maker-ptg000529l-snap-gene-2.33-mRNA-1	*MCF2*				√		√	proto-oncogene DBL
3	maker-ptg000332l-snap-gene-16.114-mRNA-1	*RPS6KA3*				√		√	ribosomal protein S6 kinase alpha-3
3	maker-ptg000904l-snap-gene-5.150-mRNA-1	*MSI1*				√		√	RNA-binding protein Musashi homolog 1
2	maker-ptg000465l-snap-gene-5.82-mRNA-1	*SLFN13*				√		√	schlafen family member 13
3	maker-ptg000253l-snap-gene-62.60-mRNA-1	*SIGLEC1*				√		√	sialoadhesin
3	maker-ptg002239l-snap-gene-7.21-mRNA-1	*SIK3*				√		√	SIK family kinase 3
3	maker-ptg000438l-est_gff_est2genome-gene-1.7-mRNA-1	*SLAMF9*				√		√	SLAM family member 9
3	snap_masked-ptg002330l-processed-gene-0.23-mRNA-1	*SMAD3*				√		√	SMAD family member 3
3	maker-ptg001574l-snap-gene-0.12-mRNA-1	*SLC8A1*				√		√	sodium/calcium exchanger 1
3	maker-ptg000090l-augustus-gene-0.0-mRNA-1	*TENM2*				√		√	teneurin-2

Supplementary Table 3. Genes with fixed alleles in hybrids that showed *com* expression proportion change over 0.1 between juveniles and adults. FC indicates Fold change in the pairwise comparison among pure bred species and hybrids, nd indicates fold change ≤|0.1| .

ID	Gene	In group 2, 3 or 6?	Blast	*com* proportion of juvenile					*com* proportion of adult					Log2FC of pairwise comparison in juvenile		Log2FC of pairwise comparison in adult		average *com* proportion of juveniles	average *com* proportion of adults	*com* proportion change
				**J1**	**J2**	**J3**	**J4**	**J5**	**A1**	**A2**	**A3**	**A4**	**A5**	***com* vs hybrid**	***rhy* vs hybrid**	***com* vs hybrid**	***rhy* vs hybrid**			
maker-ptg000051l-snap-gene-130.10-mRNA-1	*TULP4*		tubby-related protein 4	0.64	0.58	0.49	0.63	0.66	0.39	0.59	0.66	0.28	0.52	n.d.	n.d.	n.d.	n.d.	0.60	0.49	-0.11
maker-ptg000057l-augustus-gene-21.17-mRNA-1	*KLHL30*		kelch like family member 30	0.70	0.65	0.70	0.68	0.35	0.60	0.45	0.50	0.39	0.50	n.d.	n.d.	n.d.	n.d.	0.61	0.49	-0.13
maker-ptg000057l-snap-gene-35.19-mRNA-1	*NCL*		nucleolin	0.44	0.55	0.45	0.46	0.48	0.44	0.18	0.55	0.49	0.20	n.d.	n.d.	n.d.	n.d.	0.48	0.37	-0.11
maker-ptg000059l-snap-gene-7.59-mRNA-1	*TMOD4*		tropomodulin 4	0.57	0.55	0.60	0.56	0.60	0.40	0.50	0.52	0.53	0.46	n.d.	n.d.	n.d.	n.d.	0.58	0.48	-0.10
maker-ptg000085l-snap-gene-5.130-mRNA-1	*DUSP7*		dual specificity phosphatase 7	0.35	0.38	0.29	0.17	0.36	0.40	0.34	0.33	0.59	0.54	n.d.	n.d.	n.d.	n.d.	0.31	0.44	0.13
maker-ptg000113l-augustus-gene-2.31-mRNA-1	*OGT*		O-linked N-acetylglucosamine (GlcNAc) transferase	0.49	0.44	0.60	0.69	0.59	0.47	0.36	0.46	0.48	0.45	n.d.	n.d.	n.d.	n.d.	0.56	0.44	-0.12
maker-ptg000120l-snap-gene-2.54-mRNA-1	*SYNGR3*		synaptogyrin-3	0.82	0.77	0.82	0.88	0.95	0.61	0.63	0.55	0.61	0.62	**-3.41**	**1.45**	**-1.27**	n.d.	0.85	0.60	-0.25
maker-ptg000135l-snap-gene-36.1-mRNA-1	*FGD5*		FYVE, RhoGEF and PH domain containing 5	0.59	0.59	0.51	0.58	0.66	0.36	0.55	0.62	0.38	0.55	n.d.	n.d.	n.d.	n.d.	0.59	0.49	-0.10
maker-ptg000173l-snap-gene-11.29-mRNA-1	*PKD1*		polycystin 1, transient receptor potential channel interacting	0.38	0.41	0.42	0.40	0.59	0.65	0.51	0.58	0.56	0.46	n.d.	n.d.	n.d.	n.d.	0.44	0.55	0.11
maker-ptg000173l-snap-gene-4.15-mRNA-1	*DNM2*		dynamin-2	0.38	0.40	0.23	0.48	0.41	0.70	0.42	0.34	0.38	0.56	n.d.	n.d.	n.d.	n.d.	0.38	0.48	0.10
maker-ptg000265l-est_gff_est2genome-gene-6.33-mRNA-1	*KCNJ2*	2	inward rectifier potassium channel 2	0.31	0.23	0.35	0.20	0.40	0.27	0.22	0.20	0.14	0.17	n.d.	**2.40**	**-2.24**	**1.80**	0.30	0.20	-0.10
maker-ptg000312l-snap-gene-7.122-mRNA-1	*PLEKHA8*		pleckstrin homology domain containing A8	0.27	0.34	0.36	0.46	0.58	0.40	0.61	0.49	0.58	0.61	n.d.	n.d.	n.d.	n.d.	0.40	0.54	0.14
maker-ptg000332l-augustus-gene-12.62-mRNA-1	*NLRP12*		NACHT, LRR and PYD domains-containing protein 12	0.67	0.77	0.64	0.56	0.53	0.42	0.55	0.37	0.29	0.54	n.d.	n.d.	n.d.	n.d.	0.63	0.43	-0.20
maker-ptg000335l-snap-gene-4.111-mRNA-1	*KIF13B*		kinesin family member 13B	0.76	0.62	0.70	0.59	0.57	0.68	0.82	0.76	0.73	0.79	n.d.	n.d.	n.d.	n.d.	0.65	0.76	0.11
maker-ptg000399l-augustus-gene-13.63-mRNA-1	*HIP1*	3	huntingtin-interacting protein 1-related protein	0.65	0.54	0.70	0.67	0.67	0.47	0.52	0.50	0.45	0.61	n.d.	n.d.	n.d.	n.d.	0.64	0.51	-0.13
maker-ptg000425l-snap-gene-5.182-mRNA-1	*B4GALNT1*		beta-1,4-N-acetyl-galactosaminyltransferase 1	0.51	0.54	0.52	0.61	0.60	0.42	0.37	0.42	0.53	0.51	n.d.	n.d.	n.d.	n.d.	0.56	0.45	-0.11
maker-ptg000439l-augustus-gene-0.53-mRNA-1	*CD34*		hematopoietic progenitor cell antigen CD34	0.72	0.75	0.86	0.81	0.57	0.37	0.47	0.82	0.62	0.62	**-1.25**	**2.50**	**-2.16**	n.d.	0.74	0.58	-0.16
maker-ptg000457l-augustus-gene-26.21-mRNA-1	*STK10*		serine/threonine-protein kinase 10	0.53	0.64	0.59	0.58	0.53	0.59	0.62	0.43	0.30	0.33	n.d.	n.d.	n.d.	n.d.	0.57	0.45	-0.12
maker-ptg000457l-augustus-gene-7.28-mRNA-1	*HIF1AN*		hypoxia inducible factor 1 alpha subunit inhibitor	0.36	0.32	0.56	0.56	0.58	0.64	0.46	0.41	0.74	0.68	n.d.	n.d.	n.d.	n.d.	0.47	0.59	0.11
maker-ptg000465l-augustus-gene-17.6-mRNA-1	*PACSIN3*		protein kinase C and casein kinase substrate in neurons 3	0.64	0.56	0.38	0.37	0.54	0.28	0.28	0.56	0.50	0.24	n.d.	n.d.	n.d.	n.d.	0.50	0.37	-0.12
maker-ptg000469l-snap-gene-7.90-mRNA-1	*PABPC3*		immune-associated nucleotide-binding protein 3	0.53	0.62	0.52	0.60	0.49	0.53	0.46	0.41	0.44	0.38	n.d.	n.d.	n.d.	n.d.	0.55	0.45	-0.11
maker-ptg000544l-snap-gene-0.7-mRNA-1	*OSBPL1A*		oxysterol-binding protein-related protein 1	0.24	0.40	0.58	0.39	0.20	0.18	0.19	0.42	0.20	0.23	**1.41**	n.d.	n.d.	**-2.78**	0.36	0.25	-0.12
maker-ptg000568l-snap-gene-0.7-mRNA-1	*CHMP1B*		PREDICTED: Megalops cyprinoides charged multivesicular body protein 1b	0.45	0.35	0.42	0.43	0.49	0.63	0.69	0.42	0.64	0.44	n.d.	n.d.	n.d.	n.d.	0.43	0.56	0.14
maker-ptg000598l-snap-gene-3.38-mRNA-1	*ANKRD11*		ankyrin repeat domain 11	0.42	0.45	0.39	0.38	0.46	0.54	0.52	0.46	0.72	0.47	n.d.	n.d.	n.d.	n.d.	0.42	0.54	0.12
maker-ptg000600l-snap-gene-6.29-mRNA-1	*RNF25*		ring finger protein 25	0.76	0.50	0.34	0.63	0.66	0.60	0.42	0.54	0.28	0.35	n.d.	n.d.	n.d.	n.d.	0.58	0.44	-0.14
maker-ptg000702l-augustus-gene-2.13-mRNA-1	*CCDC43*		coiled-coil domain containing 43	0.60	0.43	0.53	0.57	0.50	0.44	0.30	0.55	0.31	0.41	n.d.	n.d.	**-1.30**	n.d.	0.53	0.40	-0.12
maker-ptg000771l-snap-gene-5.64-mRNA-1	*RANBP2*		E3 SUMO-protein ligase RanBP2	0.60	0.41	0.69	0.60	0.63	0.39	0.59	0.29	0.35	0.60	n.d.	n.d.	n.d.	n.d.	0.59	0.44	-0.14
maker-ptg000869l-snap-gene-17.3-mRNA-1	*SPAG9*		sperm associated antigen 9	0.36	0.40	0.45	0.48	0.43	0.47	0.43	0.64	0.51	0.57	n.d.	n.d.	n.d.	n.d.	0.42	0.53	0.11
maker-ptg000881l-augustus-gene-0.121-mRNA-1	*FILIP1*		filamin-A-interacting protein 1	0.54	0.55	0.39	0.58	0.55	0.39	0.39	0.39	0.40	0.55	n.d.	n.d.	n.d.	n.d.	0.52	0.42	-0.10
maker-ptg000908l-augustus-gene-0.6-mRNA-1	*SIGMAR1*		sigma non-opioid intracellular receptor 1	0.59	0.67	0.34	0.61	0.33	0.70	0.59	0.62	0.56	0.61	n.d.	n.d.	n.d.	n.d.	0.51	0.58	0.11
maker-ptg001156l-snap-gene-19.88-mRNA-1	*RANBP9*		RAN binding protein 9	0.62	0.52	0.49	0.53	0.45	0.46	0.52	0.33	0.37	0.28	n.d.	n.d.	n.d.	n.d.	0.52	0.39	-0.13
maker-ptg001188l-snap-gene-6.0-mRNA-1	*SCN4AA*		sodium channel protein type 4 subunit alpha A	0.48	0.54	0.60	0.56	0.59	0.68	0.68	0.61	0.64	0.71	n.d.	**-1.51**	**1.38**	n.d.	0.55	0.67	0.11
maker-ptg001248l-augustus-gene-3.147-mRNA-1	*SCARF1*		scavenger receptor class F member 1	0.75	0.48	0.68	0.61	0.72	0.57	0.48	0.71	0.56	0.44	n.d.	**-1.20**	n.d.	n.d.	0.65	0.55	-0.10
maker-ptg001309l-est_gff_est2genome-gene-5.122-mRNA-1	*CUNH10ORF71*		chromosome unknown C10orf71 homolog	0.49	0.59	0.56	0.43	0.45	0.43	0.51	0.26	0.43	0.35	n.d.	**-1.88**	n.d.	n.d.	0.52	0.39	-0.11
maker-ptg001894l-augustus-gene-1.273-mRNA-1	*HEPH1*		pannexin 1	0.35	0.66	0.45	0.43	0.55	0.61	0.71	0.67	0.53	0.47	n.d.	**1.26**	n.d.	n.d.	0.49	0.60	0.11
maker-ptg001954l-snap-gene-1.51-mRNA-1	*TTC1*		tetratricopeptide repeat domain 1	0.40	0.49	0.51	0.34	0.38	0.53	0.67	0.72	0.68	0.44	n.d.	n.d.	n.d.	n.d.	0.42	0.61	0.18
maker-ptg002126l-augustus-gene-12.96-mRNA-1	*SRP68*		signal recognition particle 68	0.56	0.58	0.41	0.50	0.66	0.67	0.74	0.68	0.44	0.76	n.d.	n.d.	n.d.	n.d.	0.54	0.66	0.12
snap_masked-ptg000207l-processed-gene-2.200-mRNA-1	*CYFIP1*		cytoplasmic FMR1 interacting protein 1	0.55	0.36	0.40	0.39	0.48	0.64	0.47	0.64	0.46	0.50	n.d.	n.d.	n.d.	n.d.	0.44	0.54	0.11
snap_masked-ptg002234l-processed-gene-0.17-mRNA-1	*PPP1R3A*	3	protein phosphatase 1 regulatory subunit 3A	0.48	0.54	0.54	0.59	0.57	0.36	0.54	0.43	0.33	0.39	n.d.	n.d.	n.d.	**-1.51**	0.54	0.41	-0.13
